# Overall survival prediction of non-small cell lung cancer by integrating microarray and clinical data with deep learning

**DOI:** 10.1038/s41598-020-61588-w

**Published:** 2020-03-13

**Authors:** Yu-Heng Lai, Wei-Ning Chen, Te-Cheng Hsu, Che Lin, Yu Tsao, Semon Wu

**Affiliations:** 1 Department of Chemistry, Chinese Culture University, Taipei, 11114 Taiwan; 2 Department of Electrical Engineering, National Tsing Hua University, Hsinchu, 30013 Taiwan; 3 Department of Electrical Engineering and Graduate Institute of Communication Engineering, National Taiwan University, Taipei, 10617 Taiwan; 4 Research Center for Information Technology and Innovation, Academia Sinica, Taipei, 11114 Taiwan; 5 Department of Life Science, Chinese Culture University, Taipei, 11114 Taiwan

**Keywords:** Non-small-cell lung cancer, Regulatory networks, Prognostic markers, Non-small-cell lung cancer

## Abstract

Non-small cell lung cancer (NSCLC) is one of the most common lung cancers worldwide. Accurate prognostic stratification of NSCLC can become an important clinical reference when designing therapeutic strategies for cancer patients. With this clinical application in mind, we developed a deep neural network (DNN) combining heterogeneous data sources of gene expression and clinical data to accurately predict the overall survival of NSCLC patients. Based on microarray data from a cohort set (614 patients), seven well-known NSCLC biomarkers were used to group patients into biomarker- and biomarker+ subgroups. Then, by using a systems biology approach, prognosis relevance values (PRV) were then calculated to select eight additional novel prognostic gene biomarkers. Finally, the combined 15 biomarkers along with clinical data were then used to develop an integrative DNN via bimodal learning to predict the 5-year survival status of NSCLC patients with tremendously high accuracy (AUC: 0.8163, accuracy: 75.44%). Using the capability of deep learning, we believe that our prediction can be a promising index that helps oncologists and physicians develop personalized therapy and build the foundation of precision medicine in the future.

## Introduction

Lung cancer is the worldwide leading cause of cancer-related mortality, with non-small cell lung cancer (NSCLC) accounting for approximately 85% of all lung cancer patients^1^. The most common NSCLC subtypes are adenocarcinoma (ADC), squamous cell carcinoma (SQC), and large cell carcinoma. Although the overall 5-year survival rate of patients diagnosed with stage I ADC was 63%, nearly 35% of patients relapsed after surgery with a poor prognosis^2^. Adjuvant treatments have been considered ideal for ADC patients with the highest risk of recurrence or death to increase survival rates^3^. Therefore, prognostic stratification is crucial for categorizing patients to help doctors make decisions on therapeutic strategies.

Recently, researchers have developed predictive methods based on gene expression profiles to classify lung cancer patients with distinct clinical outcomes, including relapse and overall survival^4^. Previous studies have shown the importance of biomarkers for NSCLC, such as *EPCAM*, *HIF1A*, *PKM*, *PTK7*, *ALCAM*, *CADM1*, and *SLC2A1*, which were used as a single biomarker for predicting prognostic condition or metastasis^5–11^. However, cancer is a systemic disease with complicated and illusive mechanisms that often involves multiple genes and cross-talk between pathways. Therefore, extending our understanding of NSCLC via the single gene biomarkers by studying the interactions between genes is essential for more accurate prognostic prediction.

Machine learning algorithms are powerful tools that apply input features (biomarkers) to capture the complicated interdependencies between these features to accurately predict clinical outcomes^12^. In addition, predicting cancer prognosis can be improved by appropriately modeling the interactions between biomarkers compared with the single biomarker approach^13^. Deep learning has seen unprecedented success in many fields, such as image recognition^14^, speech recognition^15^, and biology^16^. A deep neural network (DNN) is composed of non-linear modules, which represent multiple levels of abstraction^17^. Each representation can be transformed into a slightly more abstract level, leading to even more involved interactions among features. As a result, deep learning algorithms can extract high-level abstractions from different types of data sources and provide superior performance compared with traditional machine learning methods^18^. Thanks to the representation of features in hidden layers, a DNN can easily combine the networks for different modalities. Therefore, we aimed to propose an integrative DNN that combines both gene expression and clinical data to improve prognostic prediction for NSCLC patients.

During the flourishing development of deep learning algorithms in biomedical applications, two important works have drawn our attention. Coudray *et al*. trained their convolutional neural network (CNN) with the whole-slide images for NSCLC patients obtained from The Cancer Genome Atlas (TCGA) to classify them into adenocarcinoma (LUAD), squamous cell carcinoma (LUSC), or normal lung tissues. They suggested that deep learning models can help in detecting cancer subtypes and gene mutation^19^. Bo He *et al*. performed feature extraction on computed tomography (CT) images of NSCLC patients and built a random forest classifier for predicting the survival status^20^. Note that both their prediction models dealt with image data while we focused on gene expression and clinical information. We believe that their works and ours are complementary to each other. From the gene expression data, we selected a set of 15 prognostic biomarkers that can identify patients with adverse prognosis in the early stages. We can draw biological insights from the molecular mechanisms among them for a better understanding of the formation and metastasis of cancer cells. We motivate our systems biology feature selection process in the paragraphs below.

Due to the relative small sample size of patient data compared to the large number of genetic features, scientists have assiduously focused on feature selection algorithms that aim to obtain a subset of significantly representative features^21^. However, while traditional feature selection methods are often based on the statistical or predictive performance of the patient dataset, biological concepts were rarely considered when isolating potential gene features. Therefore, the predicted gene features to apply and improve therapeutic strategies for cancer patients are limited.

Systems biology is computational and mathematical modeling of complex biological systems that has been widely applied^22^. There is increasing interest in applying systems biology approaches to identify cancer-associated genes as feature selection strategies^23^. In this study, we established a systems biology approach for NSCLC patients, which identified eight novel survival-related genes based on seven previously well-known biomarkers. The combined 15 biomarkers were used in the following DNN model to predict the survival status of patients. Through a bimodal deep learning approach, we combined gene expression profiles and clinical data sources to predict the 5-year overall survival of NSCLC patients. We believe our significant improvements to predicting prognostic outcomes for lung cancer patients may help oncologists and physicians make accurate and precise decisions on appropriate treatment for individual patients, which may build the foundation for future personalized therapeutic strategies.

## Results

**Figure 1:**
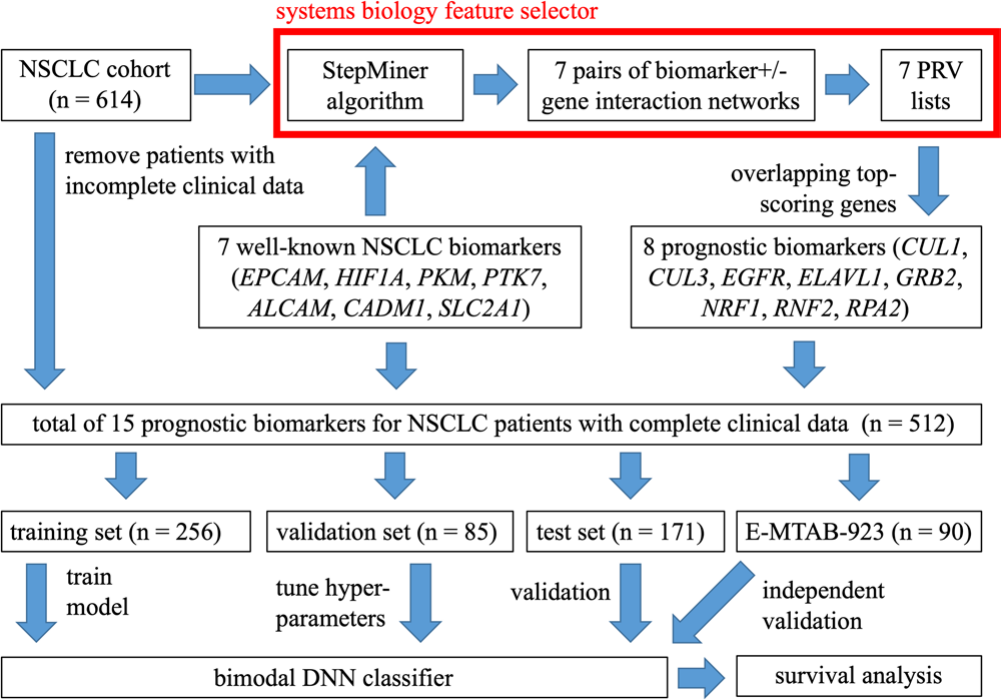
Schematic of the study design. We built 7 pairs of biomarker+ and biomarker- gene interaction networks for patients divided with high and low well-known biomarkers expression levels identified with SetpMiner, respectively. Overlapping these 7 prognosis relevance values (PRV) lists produced 8 prognostic biomarkers. We chose lung adenocarcinoma (ADC) patients with complete clinical data (n = 512) and divided them into the training (n = 256), test (n = 171), and validation (n = 85) sets. We trained deep neural networks (DNNs) using the training set and tuned hyper-parameters using the validation set. After training the DNNs, we classified the test set and conducted the survival analysis.

We integrated systems biology and deep learning approaches to predict the survival status of NSCLC patients. In addition, the systems biology approach was specifically used to identify prognostic biomarkers (gene features). The selected prognostic biomarkers were used as input features for our DNN prediction based on the 5-year survival of lung cancer patients. Moreover, we further integrated their clinical background via an integrative DNN model to improve the performance of our prediction. The schematic of our strategy is shown in Fig. 1.

### Identification of eight prognostic biomarkers for feature selection

We referred to seven well-known biomarkers that are highly correlated with NSCLC according to previous studies (*EPCAM*, *HIF1A*, *PKM*, *PTK7*, *ALCAM*, *CADM1*, and *SLC2A1*)^5–11,24,25^. Based on each of these seven biomarkers, we separated the 614 ADC patients into biomarker- and biomarker+ subgroups according to expressions by StepMiner^26^ (Fig. 1, systems biology feature selector).

We constructed interaction networks for both biomarker- and biomarker+ subgroups, resulting in a pair of gene interaction networks for each NSCLC biomarker. From each pair of interaction networks, genes were ranked by PRV (see *Methods*). We selected the top 30 genes as candidates for each well-known NSCLC biomarker. The details of the PRV for each biomarker are listed in the *Supplementary Material*. To guarantee robustness, we overlapped the seven PRV lists and found that genes, including *COPS5*, *CUL1*, *CUL3*, *EGFR*, *ELAVL1*, *GRB2*, *HSP90AA1*, *NRF1*, *PPP1CA*, *RNF2*, *RPA2*, *SIRT7*, and *SUMO1* were overlapped in all seven lists. By filtering these genes based on significance for survival (p < 0.01), the final eight prognostic biomarkers, *CUL1*, *CUL3*, *EGFR*, *ELAVL1*, *GRB2*, *NRF1*, *RNF2*, and *RPA2*, were identified. Combined with seven well-known biomarkers, a set of 15 prognostic gene biomarkers were adopted in the following analyses.

### Integrating gene expression and clinical data using a DNN

We used a DNN to exploit the interdependencies of the 15 selected prognostic biomarkers, which were fed into the DNN as input features. The output of the DNN was a binary outcome of the five-year overall survival probability for the patient after the first therapeutic treatment. The optimized structure for our DNN uses four hidden layers, each with 40 neurons, with rectified linear unit (ReLU) as the activation function and Nadam as the optimizer. To verify the effectiveness of the DNN on survival classification, we compared the performance with other well-known classifiers, such as K-Nearest Neighbors (KNN), Random Forest (RF), and Support Vector Machine (SVM) by using the same prognostic biomarkers as input features^27–29^. For the KNN classifier, the Euclidean distance was used as the distance metric, and for the SVM classifier, a Gaussian radial basis function was used as the kernel function. The parameters used in KNN, RF, and SVM were all optimized based on 10-fold cross-validation of the training set. We also compared different classifiers with the molecular prognostic index (MPI)^30^. Due to the imbalance of labels across the entire dataset (n = 512; survivals = 355 and deaths = 157), the classifiers tended to classify the patients as alive. In this case, if a naive classifier classified all patients as alive, it still reached an accuracy of 0.6953. On the other hand, AUC is a much better performance metric than accuracy that takes class imbalance into account and is a thresholdless metric (details described in the *Supplementary Materials*). We found that the performance of the DNN (AUC: 0.7926, accuracy: 0.7485) was superior to all other methods in terms of AUC when trained with only microarray data. However, the accuracy of the DNN was comparable with RF (AUC: 0.7767, accuracy: 0.7544), yet higher in AUC. Furthermore, the AUC of RF was close to our DNN model, so we could compare RF and existing work as the candidate for further investigation^30^. Moreover, we trained our DNN using only clinical patient data (age, gender, and stage). In a previous study, a clinical prognostic index (CPI) risk score was defined by using clinical data^30^. We again noted that the DNN (AUC: 0.7388, accuracy: 0.6608) achieved a significantly higher AUC than CPI (AUC: 0.6460, accuracy: 0.6257) and RF (AUC: 0.6361, accuracy: 0.6784) (see *Supplementary Materials* Tables S3–5 for more information).

**Figure 2:**
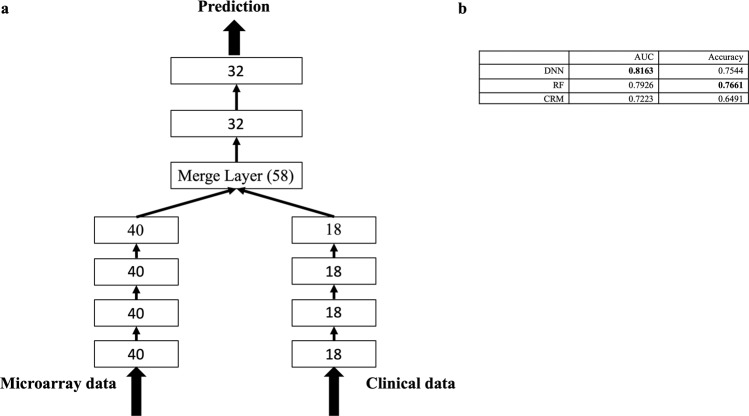
The integrative DNN structure and performance comparison with other methods. (**a**) The left branch network deals with the microarray data source and the right branch network processes the clinical data source. Both subnetworks were merged together and form an integrative network. We merged the 4th hidden layer (with 40 neurons) of the microarray DNN data and the 4th hidden layer (with 18 neurons) of the clinical DNN. The merged layer contained 58 neurons and were stacked with two hidden layers with 32 neurons each for the final prediction. (**b**) Performance comparison of the integrative DNN with other methods for combined data.

Although several studies have combined microarray and clinical data for making predictions^31^, it was difficult to integrate two heterogeneous data sources. The DNN has the flexibility to integrate heterogeneous data sources by merging the hidden layers of the neural networks. The application of a DNN for integrating different types of data sources has been successful in handling audio and video data sources^32^; however, it is relatively new to integrate gene expression and clinical data sources. Therefore, we proposed using a DNN for data integration (Fig. 2a). The weights of the integrative DNNs were trained by feeding in the microarray and clinical data simultaneously. The weights trained for the individual DNN networks were used as initial weights for the pre-training of the integrative network (see *Supplementary Materials*).

Gentles *et al*. also combined gene expression data (MPI) and clinical data (CPI) to define a composite risk model (CRM). The threshold was chosen from the median of training sets for the CRM. We compared the performances of our proposed integrative DNN with RF and CRM, as shown in Fig. 2b. The AUC performance of the integrative DNN (AUC: 0.8163, accuracy: 0.7544) was better than that of the RF (AUC: 0.7926, accuracy: 0.7661). It is important to note that after we included the clinical data, the improved AUC performance of the DNN (0.7926 to 0.8163, improved by 3%) was higher than that of the RF (0.7767 to 0.7926, improved by 2%). Our proposed integrative DNN is more powerful for integrating heterogeneous data sources reflected by the AUC performance. It is important to note that both machine learning-based algorithms significantly outperformed the CRM method (AUC: 0.7223, accuracy: 0.6491).

### From AUC to reclassification

To obtain an overall picture of the performance comparison, we also considered precision, recall, and F1-score. The F1-score is also called the F1 measure and it considers both the precision and the recall by computing the harmonic mean of precision and recall^33^. To compute these metrics, we need to find suitable cut-off points for reclassification. We used the Youden index^34^ to select cut-off points for reclassification. Such reclassification was conducted on both DNN with only microarray data and the integrative DNN with both microarray and clinical data. We calculated the cut-off points as 0.4396 and 0.4008 for the two DNNs, respectively. Similarly, we also calculated new cut-off points as 0.32 and 0.34 for the microarray RF and integrative RF, respectively. It is to note that these new cut-off points are smaller than 0.5 (original cut-off point), indicating that the number of patients classified as deaths increased after reclassification.

**Figure 3:**
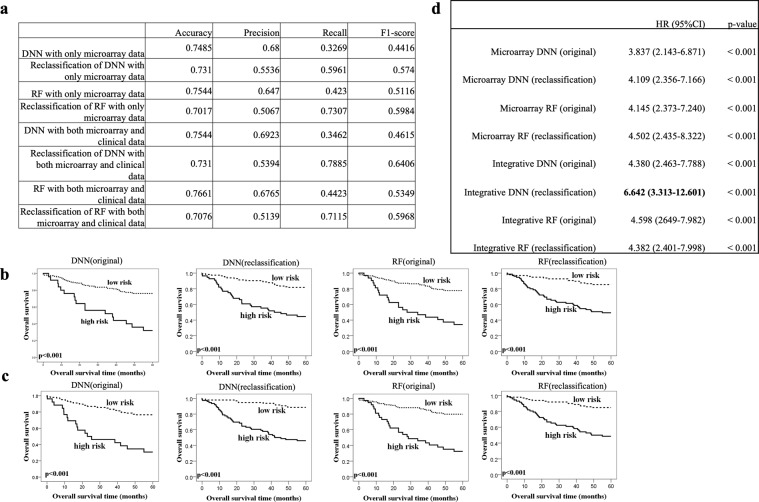
DNN and RF performance evaluation on the merged cohort. (**a**) The performance of the DNN/RF with/without reclassification with only microarray data or both microarray and clinical data. (**b**) KM analysis of overall survival in the cohort microarray test set with stratification of risk groups based on the DNN and RF trained on only the microarray data. The cut-off threshold was set at either 0.5 (original) or using the new cut-off point from Youden index (reclassification). (**c**) Both microarray and clinical data were applied to DNN and RF, and the cut-off threshold was set at either 0.5 (original) or using the new cut-off point from Youden index (reclassification). (**d**) Univariate analysis with proportional-hazards model of each classifier.

To confirm the effectiveness of reclassification, we evaluated their performances in terms of accuracy, precision, recall, and F1-score for the microarray DNN and RF and the integrative DNN and RF (Fig. 3a). Note that the imbalanced class distribution of the data made the recall and F1-score of the original classifier low. When Youden indices were used for reclassification, the evaluation criteria (recall and F1-score) improved for both the microarray and integrative DNNs and RFs. For DNNs, we observed a significant increase in recall (from 0.3269 to 0.5961 for the microarray DNN; 0.3462 to 0.7885 for the integrative DNN) and F1-score (from 0.4416 to 0.5740 for the microarray DNN; from 0.4615 to 0.6406 for the integrative DNN). Similarly, we also observed a significant increase in recall (from 0.4230 to 0.7307 for the microarray RF; 0.4423 to 0.7115 for the integrative RF) and F1-score (from 0.5116 to 0.5984 for the microarray RF; from 0.5349 to 0.5968 for the integrative RF). For RF, we observed a minor decrease in accuracy (from 0.7485 to 0.7310 for the microarray DNN; from 0.7544 to 0.7310 for the integrative DNN). Furthermore, there was a decrease in precision (from 0.68 to 0.5536 for the microarray DNN; from 0.6923 to 0.5394 for the integrative DNN), but a significant increase in recall compensates, resulting in a significant overall improvement in the F1-score. We also observed decreases in both accuracy (from 0.7544 to 0.7017 for the microarray RF; from 0.7661 to 0.7076 for the integrative RF) and precision (from 0.6470 to 0.5067 for the microarray RF; from 0.6765 to 0.5139 for the integrative RF) for RFs. Overall, the integrative DNN achieved a higher F1-score than the integrative RF, and therefore is a more desired classifier after reclassification.

### Survival analysis

To validate the effectiveness of our proposed approach, we conducted survival analysis for patients on our prognostic biomarkers and deep learning models. We divided the patients into high risk (which was predicted as dead) and low risk (which was predicted as survived) by our proposed microarray and integrative DNNs, respectively. KM analysis (see *Methods*) and proportional-hazards model were used to evaluate the results for both DNN models with/without reclassification (Fig. 3). We observed that reclassification indeed separates the two risk groups further apart based on KM analysis for both our DNN models (Fig. 3b for the microarray DNN; Fig. 3c for the integrative DNN). An improvement can also be observed in the proportional-hazards model. The microarray DNN (original) separates patients into high and low risk groups (HR: 3.837, 95% CI: 2.143–6.871; p-value < 0.001). After reclassification, we observed a more eminent separation between the two risk groups (HR: 4.109, 95% CI: 2.356–7.166: p-value < 0.001). For the integrative DNN, the separation between the two groups becomes even more significant with reclassification (HR: 6.642, 95% CI: 3.313–12.601, p-value < 0.001). We observed similar results for RFs; however, the separation of the low and high-risk groups was greater for DNNs than RFs. We can also observe that the integrative DNN achieves the highest hazard ratio, indicating that the integrative DNN is capable of extracting useful information from heterogeneous data types (Fig. 3d).

### Independent validation set

**Figure 4:**
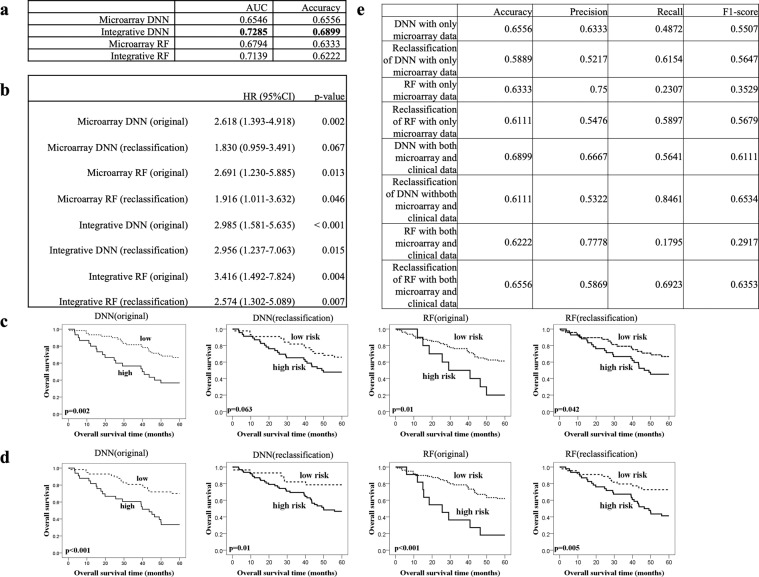
DNN and RF performance evaluation on the independent validation dataset. (**a**) AUCs and accuracies of the DNN and RF on the independent validation set. (**b**) Univariate analysis with proportional-hazards model of each classifier. (**c**) KM analysis of overall survival in the independent validation set with stratification of risk groups based on the DNN and RF trained with only the microarray data. The cut-off threshold was set at either 0.5 (original) or using the new cut-off point from Youden index (reclassification). (**d**) Both microarray and clinical data were applied to DNN and RF, and the cut-off threshold was set at either 0.5 (original) or using the new cut-off point from Youden index (reclassification). (**e**) Additional performance metrics of the DNN with/without reclassification with only microarray data or both microarray and clinical data.

To further validate the robustness of our proposed DNN models, we evaluated their performances on an independent dataset (E-MTAB-923). The patients for E-MTAB-923 (n = 90) are more balanced (51 survivals, 39 deaths) than the original cohort. We compared our proposed DNN models with RF in terms of AUC and accuracy (Fig. 4a). Interestingly, the integrative DNN outperformed RF not only in AUC, but also in accuracy. This suggests that our proposed integrative DNN model generalized better to the independent validation set. For reclassification, we used the same set of cut-off points as those in the previous section for the microarray and integrative DNNs and RFs. Since the patients of this independent validation set are more balanced than the original cohort, the results of classification are no longer extremely biased towards the low risk group. We further compared the accuracy, precision, recall, and F1-score of the microarray DNN and RF and the integrative DNN and RF (Fig. 4e). Due to different label distributions between the training set and the independent validation set, we observed that reclassification lowered the accuracies of both DNNs (from 0.6556 to 0.5889 for the microarray DNN; from 0.6899 to 0.6111 for the integrative DNN). However, reclassification improved the recall and F1-score in both DNNs. We had similar results for RFs. For RFs, we also observed an increasing trend in recall (from 0.2307 to 0.5897 for the microarray RF; 0.1795 to 0.6923 for the integrative RF) and F1-score (from 0.3529 to 0.5679 for the microarray RF; from 0.2917 to 0.6353 for the integrative RF). Although reclassification decreased the accuracies, it also improved the F1-scores. The integrative DNN model has the best F1-score and AUC in the independent validation set.

We divided the independent validation set into two risk groups and used the proportional-hazards model (Fig. 4b) and KM estimator (Figs. 4c,d) for survival analysis. Although the stratifications did not benefit from adding clinical data, improvement was observed for the proportional-hazards model. The microarray DNN (original) separated patients into high and low risk groups (HR: 2.618, 95% CI: 1.393–4.918; p-value = 0.002). After reclassification, we observed that the separation between the two risk groups was closer (HR: 1.830, 95% CI: 0.959–3.491; p-value = 0.067). For the integrative DNN, the separation between the two groups becomes even more eminent (HR: 2.985, 95% CI: 1.581–5.635; p-value < 0.001). On the other hand, we observed a moderate separation for the microarray RF (original) (HR: 2.691, 95% CI: 1.230–5.885; p-value = 0.013). After including both microarray and clinical data, we observed a more significant separation between the two risk groups (HR: 3.416, 95% CI: 1.492–7.824; p-value = 0.004). We expected the results from survival analysis to be worse since the reclassification reduced the accuracy. Recall that both DNNs was improved, although the separation between the two groups was still not eminent after reclassification.

## Discussion

Traditionally, feature selection methods can be categorized into three different types: the filter, wrapper, and embedded methods^35^. The strategy for the filter method was based on ranking the performance or mutual information of each feature, which was widely used in many domains because of their simplicity. The wrapper method worked as a black box by selecting features based on the performance of the classifier, which often showed great computational complexity and only worked well in limited classifiers. The embedded method was similar to the wrapper method; however, it focuses on reducing the amount of computational time required. All of the methods above aimed to select the top-ranked features purely based on statistical or predictive performance and disregarded the biological meaning of the gene features. Therefore, the selected gene features often lack biological insights and cannot be applied to further experimental validation.

To address this issue, we demonstrated a systems biology approach to select biologically meaningful gene features and identify eight prognostic biomarkers for NSCLC patients. These prognostic biomarkers (CUL1, CUL3, EGFR, ELAVL1, GRB2, NRF1, RNF2, and RPA2) were identified by overlapping seven computed PRV lists. Among the eight prognostic biomarkers, most of them have been reported and directly relate to NSCLC in previous studies. ELAVL1 is a well-known RNA-binding protein associated with multi-carcinogenesis, such as large cell lymphoma and glioma, by modulating RNA stability^36^. Overexpression of nuclear ELAVL1 in NSCLC patients was correlated with lymph node metastasis and may serve as a potential diagnosis biomarker^37^. In addition, while nuclear ELAVL1 was important in cancer progression, cytoplasmic ELAVL1 was also identified as an independent prognostic factor for survival in NSCLC^38^. EGFR is a well-known transmembrane protein involved in controlling cell survival and tumorigenesis in many malignancies, including NSCLC^39^. Moreover, mutated and overexpressed EGFR has been reported in a myriad of NSCLC case studies^40^. Interestingly, while EGFR was recognized as a potential therapeutic target of NSCLC, its binding adaptor, growth factor receptor bound protein 2 (GRB2), was shown to be a stabilized EGFR and co-activated downstream to the MAPK/ERK pathway^40^. Among the Cullion family, Cullin 1 (CUL1) is one of the scaffold proteins in E3 ubiquitin ligase involved in cancer progression. High expression was correlated to patient survival rate, which was identified as an independent prognostic factor in NSCLC^41^. There are eight members in the Cullin family. In addition to CUL1, CUL3 is known as a scaffold protein in the ubiquitin-proteasome system and contributes to cellular regulation, such as cell cycles, protein trafficking, and stress response, which are common tumorigenesis phenomenon when mutated. Furthermore, one substrate adaptor of CUL3, kelch-like ECH-associated protein (Keap), was first identified as an inhibitor of transcriptional factor Nf-E2-related factor 2 (Nrf2) and played important roles in anti-oxidation stress and cell defense during cancer suppression^42^. Although there is only limited evidence addressing the function of Nrf1 and Nrf2 in prostate cancer^43^, the genetic and functional conservation between them identifies their active roles in lung cancer progression. Ring finger protein 2 (RNF2), a member of the group II polycomb group (PcG) protein, was highly expressed in many types of human malignancies^44^. Despite the role RNF2 plays in biological processes in cancers via its diverse mechanisms, it highlights the potential oncogenic activity of RNF2 on NSCLC. Replication protein A 2 (RPA2), a single-strand DNA binding protein, processes DNA metabolism in response to DNA damage-associated replication arrest^45^. It has also been considered a potential therapeutic target and prognostic indicator for colon cancer, as shown by differences in its immunohistochemical expression between cancer patients and controls^46^. To summarize, each biomarker we identified showed great potential for being a prognostic biomarker based on its biological background.

**Figure 5:**
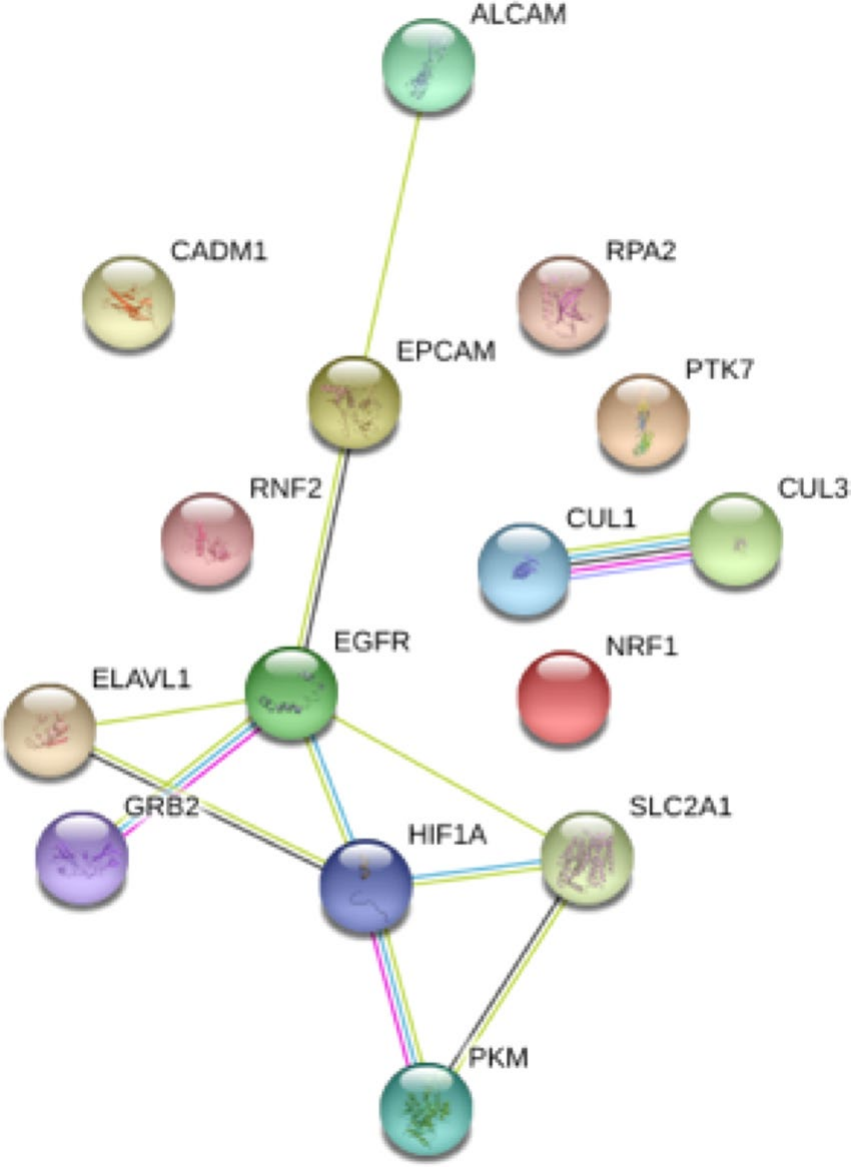
The interaction network of prognostic biomarkers. Visualization of interdependencies of the 15 selected biomarkers via STRING (https://string-db.org/).

We further analyzed eight prognostic biomarkers using GSEA (Gene-Set Enrichment Analysis)^47^. Using GO biological process enrichment analysis, we found that CUL1, CUL3, RNF2, and RPA2 overlap in their mitotic cell cycles, which has important biological implications in tumor development. We also analyzed these prognostic biomarkers by investigating their interdependencies using STRING (https://string-db.org/)^48^ (Fig. 5). The interaction between GRB2 and EGFR were experimentally verified^49^; both were characterized in the KEGG “Non-small cell lung cancer pathway” (pathway #5223). In addition, the interactions among GRB2, EGFR, HIF1A, and SLC2A1 were also highly correlated with cancer in the KEGG “Pathways in cancer” (pathway #5200).

One reason our proposed PRV feature selection method performs well in not only the test set, but also the independent validation set, is due to the robustness emphasized in our systems biology approach. Several steps were taken to ensure the robustness of our feature selection method. Firstly, we used gene expression data from six different datasets and selected only genes with the same probe number. Secondly, our PRV feature selection method was applied to each of the seven well-known NSCLC biomarkers in our data cohort. We then constructed seven different paired interaction networks to obtain seven PRV lists. The eight prognostic biomarker genes were selected via overlapping these seven PRV lists. In other words, we only selected the prognostic biomarkers that appeared in all seven PRV lists. This selection process guarantees robustness in our method based on the superior predictive performance of our proposed integrative DNN. In the future, we can not only choose well-known biomarkers from the literature, but use other clinical outcomes to group patients for selecting features.

In most of the existing work, only gene expressions were used as features for training classifiers, with the aim of predicting various disease outcomes^12,21^. In recent years, some researchers have begun to consider combining gene expression and clinical data to make such predictions^30,31^. In this study, we applied the concept of bimodal learning to construct an integrative DNN where two heterogeneous modalities (gene expression and clinical data) were integrated for predicting ADC patient overall survival. By using two modalities, the integrative DNN approach is capable of providing the missing information left by the other observed modality. Compared with our microarray DNN, we observed an increase in AUC and accuracy from the integrative DNN. We also demonstrated improved prognostic performance for survival analysis. This highlights the benefit of integrating microarray and clinical data via our integrative DNN approach. A good bimodal learning model possesses certain properties. The joint representation should be similar in its feature space, implying that the two heterogeneous data sources correspond to similar concepts. We use two DNNs to extract features from each data source and jointly train them for the complete bimodal learning model in the combined layers. From this, the combined model better integrates two data modalities into a joint representation. Furthermore, image data such as CT-scans and whole-slide images were utilized for training deep learning models to provide tissue classification or patient overall survival prediction^19,20^. We believe that our work and theirs are perfect complements to each other. For future work, we could use more than two types of data sources to construct a multimodal learning model for even more accurate prediction.

## Methods

### Microarray data preprocessing

The open access microarray data were downloaded from the National Center for Biotechnology Information (NCBI) Gene Expression Omnibus (GEO) database (http://www.ncbi.nlm.nih.gov/geo) with accession numbers GSE19188, GSE29013, GSE30219, GSE31210, GSE37745, GSE50081 with the same platform: the human Affymetrix HG-U133 Plus 2.0 platform (GPL570). Six independent GEO datasets were merged into one cohort with 614 patients. We separated 256 patients as the training set, 85 patients as the validation set, and 171 patients as the test set, all of which have complete clinical data. E-MTAB-923 was taken as an independent validation set with the same platform. Table 1 provides detailed information on the cohort. The median overall survival time is 56.25 months. All the datasets were preprocessed by the robust multi-array average (RMA) algorithm and gene expression values were log2 transformed.

**Table 1:** Clinical characteristics of patients.

	Cohort training set	Cohort validation set	Cohort test set	E-MTAB-923
No. of patients	256	85	171	90
Median of age	64 (32–84)	64 (44–86)	63 (32–86)	65 (35–84)
Male	126 (49.8%)	47 (55.3%)	93 (54.4%)	14 (15.6%)
Female	130 (50.2%)	38 (44.7%)	78 (45.6%)	76 (84.4%)
Stage IA	131	39	81	28
Stage IB	83	32	61	26
Stage IIA	6	1	6	2
Stage IIB	26	8	16	7
Stage III & IV	14	5	7	27
No. of deaths	77	26	52	39

### Prognosis relevance value

Based on the gene expression for each of the well-known biomarkers, we divided patients into two different subgroups (biomarker- and biomarker+) via the StepMiner algorithm^26^. Crucial genes were identified by a systematic comparison between subgroups. For both biomarker- and biomarker+ groups, we constructed the corresponding gene interaction networks (interaction networks)^50^. According to the constructed interaction networks, we defined prognosis relevance values (PRV) to measure the difference between biomarker+ and biomarker- interaction networks for each gene (details in the Supplementary Material). Genes with a larger PRV show a significant difference among interactions or connections compared with other genes, which are potential prognostic biomarkers.

### Bimodal DNN (integrative DNN)

A DNN is composed of one input and one output layer, with many hidden layers in between representing multiple levels of abstraction. Each hidden layer is composed of many neurons. Deep learning has been successfully applied to supervised learning for combining different modalities. For our dataset, we not only used microarray data, but also clinical data. Here, we combined two DNNs (one for the microarray data input and the other for the clinical data input) by merging their output layers and further concatenating several hidden layers before reaching the final prediction. The integrative DNN can be expected to benefit from combining the two separate data sources.

### Experimental details for benchmark models

We performed 10-fold cross-validation to select hyper-parameters for the benchmark models. For KNN, we selected Euclidean distance as the distance measure and considered at most 30 nearest neighbors. We utilized RBF kernel for SVM with different levels of L2-regularization and different gamma values for the kernel. For RF, we restricted the maximum depth as one-third of the dimension of the input data dimension and varied the number of trees used. The detailed searched optimal parameters were summarized in Table S6.

### Bimodal DNN hyper-parameter selection

For bimodal DNN, we varied the number of neurons in the two DNNs for microarray and clinical data and observed their cross-entropy losses in the validation set (Table 1), as illustrated in Table S7. We found the best model by selecting the one with the least validation loss. We also performed a similar search for the best optimizer, and the results are shown in Table S8. We found the Nadam optimizer performed the best. Learning curves for pre-training the microarray and clinical subnetworks, as well as the merged bimodal network, were illustrated in Figures S2, S3, and S4, respectively. The final model structure of our DNN contained four layers with 40 neurons in each layer for the two DNN for microarray and clinical data, and Nadam optimizer and ReLU activation function was adopted with L2-regularization. The learning rate was set to 0.006, and we followed the default settings in Nadam for other parameters. Early stopping was also included throughout the training process to avoid overfitting. We limited the model to train no longer than 100 epochs with batch size 20 and stop training if the validation loss was not improved for over 30 epochs.

### From AUC to reclassification

The receiver operating characteristic^51^ curve is a graphical plot that illustrates the diagnostic ability of a binary classifier system created by plotting the true positive rates against the false positive rates at various threshold settings. The area under the ROC curve has been used for evaluating how well the model performs^51^. Under some conditions, we can achieve a better reclassification performance by adjusting the cut-off points. In this study, the cut-off points were determined with the help of Youden index^34^, which is a frequently-used summary measure of the ROC curve. In previous classification tasks, we classified a patient by comparing the probabilities of patient survival outcomes with 0.5 for all classifiers. To further improve predictive performance, new cut-off points determined with Youden index were used for reclassification.

### Survival analysis

In our study, overall survival time was calculated from the date of surgery to the date of death. We predicted the survival status of patients within 5-year. Therefore, patients were treated as alive patients when survival time was greater than five years. Survival curves were demonstrated based on a Kaplan-Meier estimation for five years and compared using a log-rank test^52^ (KM analysis). We applied the cox proportional-hazards model to analyze the relationship between the prognostic genes for survival^53^. The hazard ratios (HR) and confidence intervals (CI) were reported.

## Supplementary information

Supplementary Materials.

## Data Availability

All data generated or analyzed during this study are included in this published article and its supplementary information files.

## Code availability

Model source codes are available at https://github.com/idssplab/overall_survival_nsclc.
