# Supplementary material for: Overall survival prediction of non-small cell lung cancer by integrating microarray and clinical data with deep learning
Source: Sci Rep. 2020 Mar 13;10:4679. doi: 10.1038/s41598-020-61588-w (PMC7069964; doi:10.1038/s41598-020-61588-w)
Supplement: Supplementary file 1 — Supplementary Materials. [file 41598_2020_61588_MOESM1_ESM.pdf]

Supplementary Material

Overall survival prediction of non-small cell lung cancer by integrating microarray and clinical data with deep learning

Yu-Heng Lai, Wei-Ning Chen, Te-Cheng Hsu, Che Lin, Yu Tsao, Semon Wu

Calculation of prognosis relevance value

Based on the gene expression of each of the seven well-known biomarkers, we could divide patients into two different subgroups, biomarker- and biomarker+, via the StepMiner algorithm to identify crucial genes using a systematic comparison between these subgroups. The goal of the StepMiner algorithm is to find a one-step function that fits the ordered samples. The StepMiner algorithm defined a "step" for a biomarker according to the largest jump of expression values from low-to-high as a threshold. To suppress noise, an intermediate region was defined as 0.5 below and 0.5 above the threshold. We then obtained two subgroups for each well-known biomarker, i.e., the biomarker- and biomarker+ groups, excluding the noise zone. For both the biomarker- and biomarker+ groups, we constructed the corresponding interaction networks. To construct the interaction network, the following dynamic model was defined for the i-th protein:

$$x_i[n] = \sum_{j=1}^{Ni} a_{ij} x_j[n] + \omega_i[n] \qquad (1)$$

where $x_i[n]$ denotes the expression levels for the i-th gene at sample n; a_{ij} denotes the interactions ability between the i-th gene and its j-th interacting gene; $\mathrm{N_i}$ is the total number of interactions with the i-th gene; and $\omega_i[n]$ represents stochastic noise. In order to prune the false positive interactions, we applied the Akaike information criterion (AIC) model order selection and t-test to reveal the number of valid associations and determine the model parameters.

The dynamic model for the interaction network in (1) can be rewritten as:

$$X(n) = AX(n) + \omega(n) \qquad (2)$$

where

$$X(n)=\begin{bmatrix} x_1[n] \\ x_2[n] \\ \vdots \\ x_M[n] \end{bmatrix},\ A=\begin{bmatrix} a_{11} & \cdots & a_{1M} \\ \vdots & a_{ij} & \vdots \\ a_{M1} & \cdots & a_{MM} \end{bmatrix},\ W(n)=\begin{bmatrix} \omega_1[n] \\ \omega_2[n] \\ \vdots \\ \omega_M[n] \end{bmatrix}$$

where M is the number of interacting genes in the refined interaction network; matrix A denotes the interactions ability matrix; and $W(n)$ is the noise matrix.

If a_{ij} is equal to zero, then there is no interaction between genes i and j or it is identified as a false positive and pruned in the refined interaction network. For convenience, A_k^+ and A_k^- are denoted as the interaction ability matrices for biomarker+ and biomarker- interaction networks, respectively:

$$A_k^+=\begin{bmatrix} a_{11,k}^+ & \cdots & a_{1M,k}^+ \\ \vdots & \ddots & \vdots \\ a_{M1,k}^+ & \cdots & a_{MM,k}^+ \end{bmatrix},\ A_k^-=\begin{bmatrix} a_{11,k}^- & \cdots & a_{1M,k}^- \\ \vdots & \ddots & \vdots \\ a_{M1,k}^- & \cdots & a_{MM,k}^- \end{bmatrix}$$

where k denotes the index for the considered well-known biomarker.

According to the constructed interaction networks, we define a matrix D_k to measure the difference between biomarker+ and biomarker- interaction networks for the k-th well-known biomarker:

$$D_k = A_k^+ - A_k^- = \begin{bmatrix} d_{11,k} & \cdots & d_{1M,k} \\ \vdots & \ddots & \vdots \\ d_{M1,k} & \cdots & d_{MM,k} \end{bmatrix} \tag{3}$$

$$= \begin{bmatrix} a_{11,k}^+ - a_{11,k}^- & \cdots & a_{1M,k}^+ - a_{1M,k}^- \\ \vdots & \ddots & \vdots \\ a_{M1,k}^+ - a_{M1,k}^- & \cdots & a_{MM,k}^+ - a_{MM,k}^- \end{bmatrix} \tag{4}$$

where $d_{ij,k}$ denotes the difference in interaction abilities between the i-th and j-th gene for the k-th well-known biomarker. To identify candidate prognostic biomarkers from D_k, the PRV is defined as

follows:

$$PRV_{i,k} = \sum_{j=1}^{M} \left| d_{ij,k} \right| \tag{5}$$

where $PRV_{i,k}$ in (5) represents the difference in interaction ability for the i-th gene and the k-th well-known biomarker.

PRV lists

Table S1: Top 30 PRV genes for seven well-known NSCLC biomarkers

EPCAM	***HIF1A***	***PKM***	***PTK7***	***ALCAM***	***CADM1***	***SLC2A1***
UBC	*UBC*	***ELAVL1***	*UBC*	*UBC*	*UBC*	*UBC*
NRF1	***NRF1***	***NRF1***	***NRF1***	***ELAVL1***	***NRF1***	***NRF1***
ELAVL1	***ELAVL1***	*APP*	***ELAVL1***	***NRF1***	***ELAVL1***	***ELAVL1***
APP	***SUMO1***	***SUMO1***	*APP*	*APP*	*APP*	*HSPA4*
SUMO1	***RNF2***	***RNF2***	***SUMO1***	***SUMO1***	***SUMO1***	***SUMO1***
CUL3	***CUL3***	***CUL3***	***CUL3***	***CUL3***	***CUL3***	*HEC W2*
RNF2	***SIRT7***	*NEDD8*	***SIRT7***	*HNRNPU*	***RNF2***	***CUL3***
COPS5	*NEDD8*	***COPS5***	*HSPA4*	*HECW2*	***COPS5***	*NEDD8*
EGFR	***EGFR***	***RPA2***	*HECW2*	***RNF2***	*YWHAQ*	***RNF2***
HSP90AA1	***GRB2***	***SIRT7***	***RPA2***	***SIRT7***	***GRB2***	*ILF3*
PARK2	***RPA2***	***GRB2***	***RNF2***	***COPS5***	***RPA2***	***COPS5***
GRB2	***COPS5***	*YWHAQ*	*NEDD8*	*NEDD8*	***SIRT7***	*HNRNPU*
HECW2	***HSP90AA1***	***HSP90AA1***	***COPS5***	*ILF3*	***EGFR***	*SIRT1*
SIRT7	*YWHAB*	***EGFR***	*CALM1*	***EGFR***	***HSP90AA1***	***HSP90AA1***
DHX9	***PPP1CA***	*PARK2*	***GRB2***	***GRB2***	*PARK2*	***SIRT7***
SRRM2	*ICT1*	***CUL1***	***HSP90AA1***	***RPA2***	*YWHAB*	***GRB2***
YWHAQ	***CUL1***	*YWHAB*	*WWOX*	***HSP90AA1***	*IGSF8*	*YWHAB*
CDKN1A	*RPA1*	*CUL5*	*HNRNPU*	***PPP1CA***	***PPP1CA***	***PPP1CA***
YWHAB	*CAND1*	*HDAC1*	*CDKN1A*	*YWHAQ*	***CUL1***	*SMAD3*
PPP1CA	*HDAC1*	*FBXO6*	*PARK2*	*SIRT1*	*HECW2*	***RPA2***
VCP	*VCP*	*HDAC2*	*YWHAQ*	*YWHAB*	*DHX9*	***EGFR***
CUL1	*CDK2*	*MDFI*	***CUL1***	***CUL1***	*CALM1*	*YWHAQ*
CALM1	*KDM1A*	*BMI1*	*EIF4A3*	*SMAD3*	*FBXO6*	*HSPA8*
H2AFX	*FN1*	*KDM1A*	*RB1*	*PARK2*	*CDKN1A*	*CREBBP*
FBXO6	*CUL5*	***PPP1CA***	*FBXO6*	*FBXO6*	*HDAC6*	***CUL1***
HDAC1	*CUL2*	*IKBKG*	*HDAC6*	*CAND1*	*HDAC1*	*PARK2*

CREBBP	*COPS6*	*HDAC3*	***PPP1CA***	*HDAC1*	*CDK2*	*HDAC6*
HDGF	*PAXIP1*	*HDAC6*	***EGFR***	*IKBKG*	*VCP*	*FBXO6*
RPA2	*ILF3*	*ICT1*	*CREBBP*	*ICT1*	*ILF3*	*HDAC1*
HNRNPU	*RPA3*	*CDK2*	*SMAD2*	*VCP*	*HDGF*	*HDAC2*

Survival analysis for the eight identified prognostic biomarkers

We further conducted survival analysis for our eight identified prognostic biomarkers (*CUL1*, *CUL3*, *EGFR*, *ELAVL1*, *GRB2*, *NRF1*, *RNF2*, and *RPA2*). Based on the expression level of each prognostic feature gene, patients were stratified into two groups, biomarker- and biomarker+, by StepMiner. The survival analysis was conducted using Kaplan-Meier methods and a univariate proportional-hazards model, as illustrated in Table S2 and Figure S1. This indicates that all of the eight prognostic feature genes were already feasible for prognosis to a certain degree. Cancer, however, is a complicated disease. It is unlikely that a single prognostic biomarker can accurately predict the clinical outcome of a cancer patient. We therefore exploited the interdependencies between the eight prognostic biomarkers combined with the original seven well-known NSCLC biomarkers via a DNN. Hopefully, we can provide a more accurate and robust approach for predicting the prognosis of NSCLC patients.

Table S2: Univariate analysis of each prognostic biomarker

	HR (95% CI)	p-value
CUL1	1.166 (0.854–1.591)	0.334
CUL3	0.761 (0.573–1.012)	0.061
EGFR	0.319 (0.227–0.448)	< 0.001
ELAVL1	0.330 (0.232–0.469)	< 0.001
GRB2	1.386 (0.683–2.814)	0.366
NRF1	0.400 (0.298–0.537)	< 0.001
RNF2	0.671 (0.506–0.890)	0.006
RPA2	1.040 (0.550–1.965)	0.904

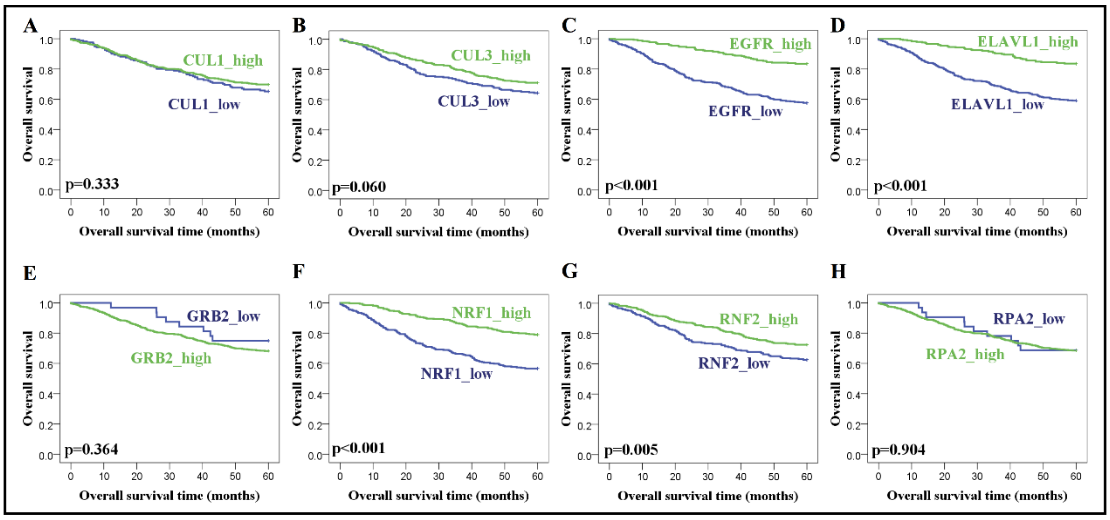

Figure S1. **KM curves of the eight prognostic biomarkers in the cohort dataset.** To study the relationship between prognostic biomarkers expression and survival, we used the StepMiner algorithm to stratify the NCBI-GEO datasets into two groups. (A–H) NSCLC patients (n = 614) were divided into two groups, which were analyzed for overall survival.

Comparison of DNN with other classifiers

The threshold was chosen from the median of the training sets for MPI. In Table S3, we compared the DNN with all the above methods by evaluating their performance on the test set. We demonstrated that the performance of the DNN (AUC: 0.7926, accuracy: 0.7485) was superior to all other methods in terms of AUC. Note that the DNN has comparable accuracy with RF (AUC: 0.7767, accuracy: 0.7544), but a higher AUC. We observed that the MPI has the worst performance both in AUC and accuracy. This indicates that our proposed framework that integrates systems biology and deep learning approaches to predict prognosis of NSCLC is more favorable than the MPI approach, even when the DNN is replaced by other well-known classifiers.

Table S3: Comparison of DNN with other methods for microarray data

	AUC	Accuracy
DNN	**0.7926**	0.7485
RF	0.7767	**0.7544**
KNN	0.7705	0.7135
SVM	0.7275	0.7018
MPI	0.7209	0.6725

We also considered different gene features, e.g., seven well-known NSCLC biomarkers, eight identified prognostic biomarkers, and nine MPI biomarkers for the microarray DNN (Table S4). We observed that irrespective of whether the classifier used seven well-known biomarkers or eight newly prognostic biomarkers as features, the predictions for both were poorer than when nine MPI biomarkers were used. However, the classifier had the best prediction when we included seven well-known biomarkers and eight newly prognostic biomarkers as features.

Table S4: Other gene features for microarray DNN

	AUC	Accuracy
Seven well-known + eight newly	0.7926	0.7485
Nine MPI biomarkers	0.7736	0.7017
Seven well-known biomarkers	0.7314	0.6959
Eight identified biomarkers	0.7233	0.6842

Training DNN using clinical data

In this section, we trained our DNN using patients' clinical data (age, gender, and stage). Hyper-parameters were tuned the same way as described in the previous section. The optimized structure for our DNN uses five hidden layers, each with 18 neurons, Relu as the activation function, and Nadam as the optimizer. Gentles et al. also defined a clinical prognostic index (CPI) risk score using patient clinical data. RF and CPI are compared in Table S5.

Table S5: Comparison of DNN with other methods for clinical data

	AUC	Accuracy
DNN	**0.7388**	0.6608
RF	0.6361	**0.6784**
CPI	0.6460	0.6257

We can again see that our DNN (AUC: 0.7388, accuracy: 0.6608) achieves significantly higher AUC than the other methods and a comparable accuracy with RF (AUC: 0.6361 accuracy: 0.6784). This may indicate that our DNN is more capable of capturing the complicated interdependencies between the features of the clinical data with the cancer survival outcome.

Details of cross-validation hyper-parameter search

We performed 10-fold cross-validation on the benchmark models while searching for the best hyper-parameters. Table S6 summarizes the detailed parameter settings throughout the search, and the optimal parameters were marked bold.

Table S6: Parameters visited in 10-CV for the benchmark models.

models	parameters visited
KNN	- distance: **Euclidian**<br>- number of neighbors: 1 ~ **30**
SVM	- kernel: radial basis function (RBF) (with bias term)<br>- regularization constant for L2 violation penalty: 1/16, 1/8, …, **4**, 8, 16<br>- gamma constant for RBF kernel: 1/16, 1/8, **1/4**, …, 1
RF	- max depth = 15/3 = **5**<br>- number of trees in the ensemble: 200, **500**, 1000

Using DNN to exploit the interdependencies of the selected prognostic biomarkers

In this section, we used a DNN to exploit the interdependencies of the 15 selected prognostic biomarkers (seven well-known and eight newly identified biomarkers). Specifically, these 15 biomarkers of a patient were fed into the DNN as input features. The output of the DNN was a binary outcome of survival probability of the patient five years after the first treatment. We used 512 ADC patients to train the DNN, of which half were randomly assigned as a training set (n = 256), a third of the samples as a test set (n = 171), and the remaining sixth as a validation set (n = 85). The DNN was determined based on the training set. After training, we optimized classification performance by

adjusting the DNN hyper-parameters (numbers of hidden layers and neurons in each hidden layer) based on the validation set. We evaluated the performance of the DNN for different hidden layers (e.g. 1, 2, 3, 4, and 5) with a different number of neurons (e.g. 8, 16, 24, 32, 40, and 48) via the validation set.

Due to the small sample size of the validation set, we obtained almost the same accuracy in several different structures, making it difficult to determine the best structure. Therefore, we instead evaluated DNN model performance using the loss function (cross entropy). We concluded that when the number of neurons is small, increasing the number of hidden layers reduces the loss function. As indicated in Table S7, the DNN structure that comprises four hidden layers, with 40 neurons per layer, achieves minimum loss. This DNN structure was used in the following experiments.

Table S7: Loss function of DNN with different numbers of layers and neurons

neurons / layers	8	16	24	32	40	48
1	0.6068	0.6115	0.5605	0.5505	0.5683	0.5544
2	0.5653	0.5791	0.5426	0.5828	0.5601	0.5503
3	0.5476	0.5626	0.5463	0.5829	0.5415	0.5564
4	0.5479	0.5957	0.5735	0.5615	**0.5361**	0.5570
5	0.5392	0.5634	0.5558	0.5556	0.5541	0.5549

We further tested the activation functions, such as Sigmoid, hyperbolic tangent (Tanh), and rectified linear unit (Relu), with different optimizers (SGD, Adam, Adamax, and Nadam) on the survival classification task of the DNN. The results are summarized in Table S8.

Table S8: Loss function of DNN with different activation functions and optimizers

	Sigmoid	Tanh	Relu
SGD	0.6125	0.5778	0.5610
Adam	0.5476	0.5885	0.5461
Nadam	0.5463	0.5525	**0.5361**
Adamax	0.5476	0.5920	0.5521

We observed that the optimizer Nadam was uniformly better than SGD for the same DNN structure for each of the tested activation functions. When we used Nadam as the preferred optimizer, the loss function was smallest for the Relu activation functions. After extensive numerical experiments, the final model structure of our DNN contained four layers, with 40 neurons in each layer, along with the Relu activation function and Nadam optimizer.

We further illustrate the binary cross-entropy loss for every epoch in Figures S2, S3, and S4 where they represent the loss for pre-training the microarray subnetwork, pre-training clinical subnetwork, and training the merged bimodal network, respectively. As shown in the figures, the losses decreased throughout the training process and our model had small generation gap from training set to testing set.

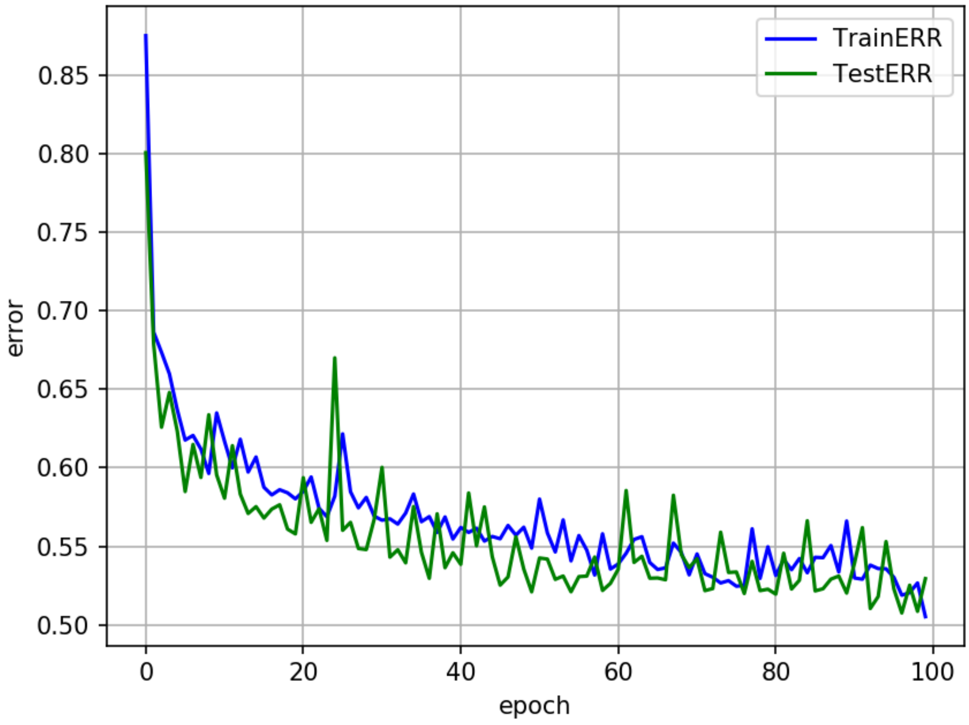

Figure S2. **Binary cross-entropy loss for pre-training microarray subnetwork for every epoch.**

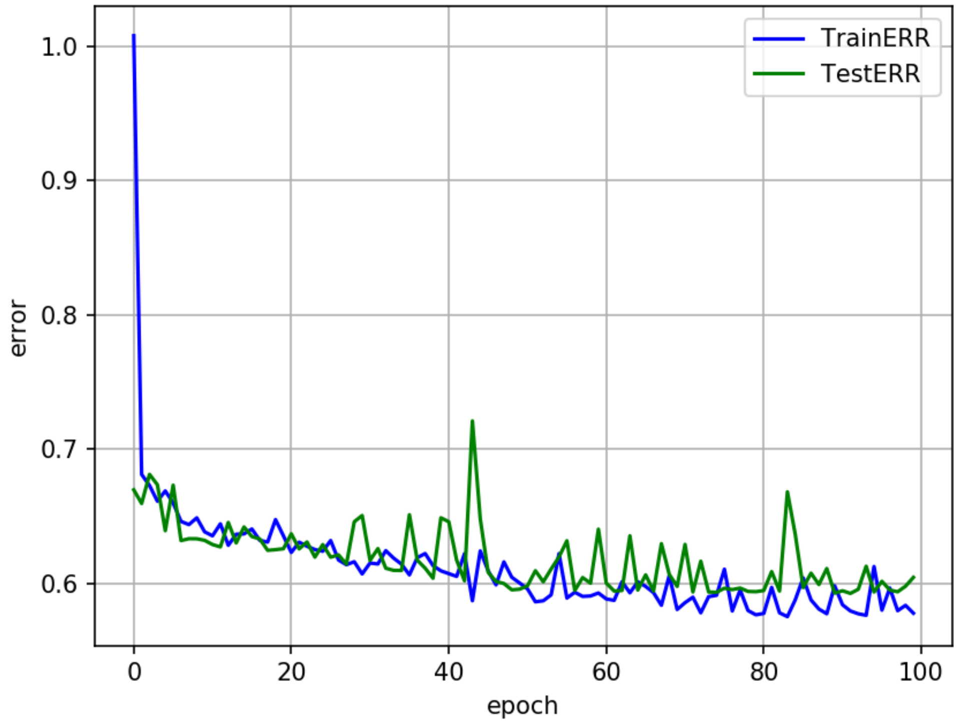

Figure S3. **Binary cross-entropy loss for pre-training clinical subnetwork for every epoch.**

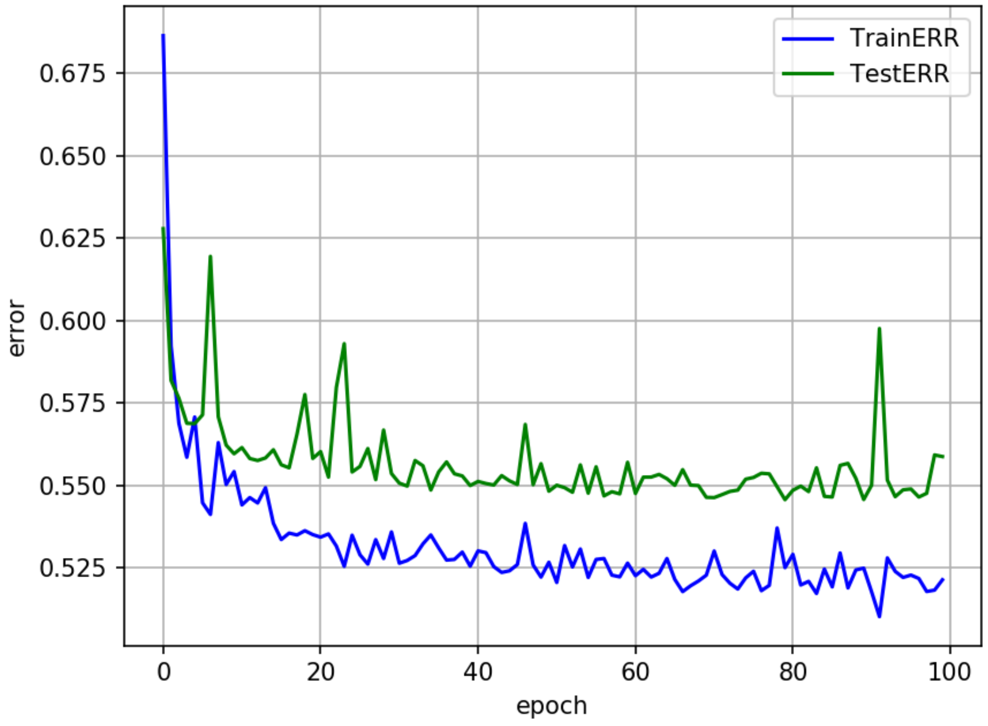

Figure S4. Binary cross-entropy loss for training merged bimodal network for every epoch.

Different data fusion methods

We also considered other data fusion methods for the DNN; Early Fusion (concatenated all features in first layer), Intermediate Fusion (bimodal learning), and Late Fusion (merging fully connected two sub-networks' output layers), as shown in Table S9. We can observe that the Intermediate Fusion method had the best performance.

Table S9: Other data fusion methods for combined DNN

	AUC	Accuracy
Early Fusion	0.8000	0.7368
Intermediate Fusion	0.8163	0.7544
Late Fusion	0.7958	0.7544

Different data partition

To confirm that our data is not limited to one special partition, we tested our model on three different data partitions. We divided the dataset into three partitions, then took the first (original), second, and third partition as test sets in each round, respectively (Table S10 and Table S11). Although the second and third results were not as good as previously shown, they still showed acceptable classification capabilities.

Table S10: Microarray DNN for 3-fold partition

	AUC	Accuracy
Original	0.7926	0.7485
Second	0.7593	0.7368
Third	0.7606	0.7000
Mean	0.7708	0.7284

Table S11: Combined DNN for 3-fold partition

	AUC	Accuracy
Original	0.8163	0.7544
Second	0.7799	0.7836
Third	0.7696	0.7294
Mean	0.7886	0.7554

Discussion of death probability for patients

We discussed probability regions of patients who had either survived or died. There are three regions ([Probability < Youden index], [Youden index < Probability < 0.5], and [0.5 < Probability]) in our discussion. We observed that it was difficult for the classifiers to classify patients in the [Youden index < Probability < 0.5] region (Table S11 and Table S12). The probability distribution is also plotted in Figure S5.

Table S12: The distribution of patients in three regions for microarray DNN in the test set.

Probability region	Survival	Death
Probability<Youden index	94	21
Youden index<Probability<0.5	17	14
0.5<Probability	8	17

Table S13: The distribution of patients in three regions for integrative DNN in the test set.

Probability region	Survival	Death
Probability < Youden index	84	11
Youden index < Probability < 0.5	27	23
0.5 < Probability	8	18

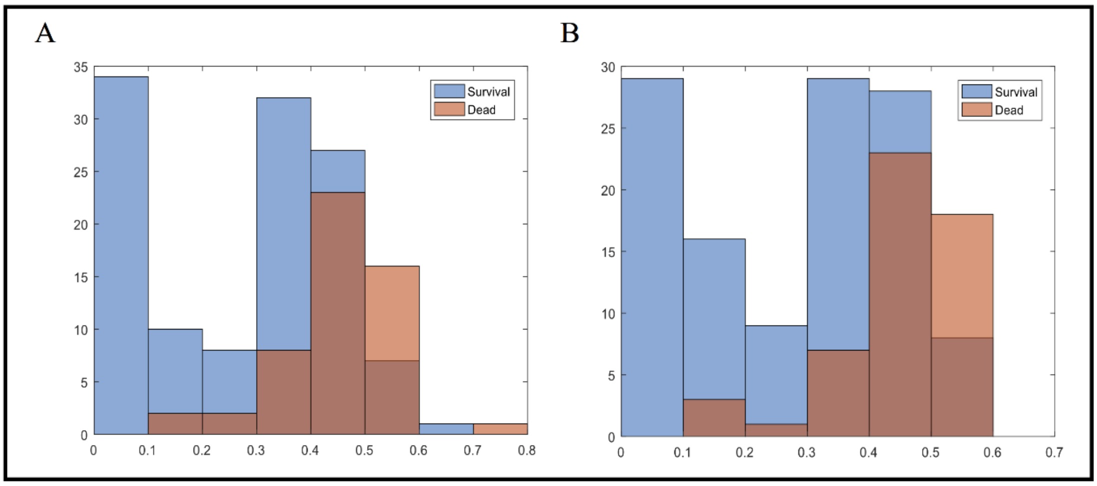

Figure S5. The probability distribution of patients in (A) microarray DNN and (B) integrative DNN in the test set.

We also found similar results for the independent validation set. We observed that two groups of probabilities (survival and death) were still not eminent in the microarray DNN. Specifically, it did not classify the [Probability < Youden index] region well in the microarray DNN. In other words, the integrative DNN was more generalizable after including the microarray and clinical data.

Table S14: The distribution of patients in three regions for microarray DNN in the independent validation set.

Probability region	Survival	Death
Probability < Youden index	29	15
Youden index < Probability < 0.5	11	5
0.5 < Probability	11	19

Table S15: The distribution of patients in three regions for integrative DNN in the independent validation set.

Probability region	Survival	Death

Probability < Youden index	22	6
Youden index < Probability < 0.5	18	11
0.5 < Probability	11	22

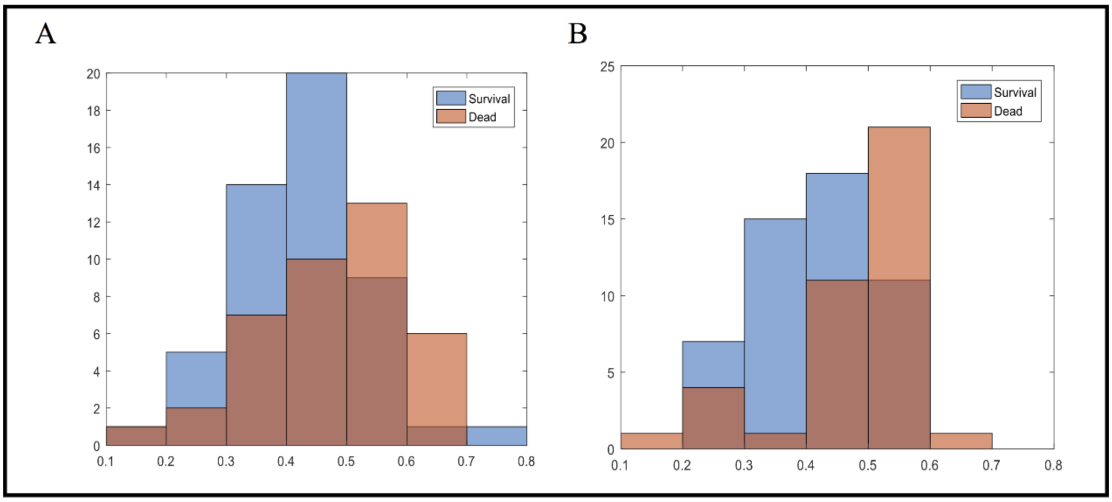

Figure S6. The probability distribution of patients in (A) the microarray DNN and (B) integrative DNN in the independent validation set.
